# Comprehensive analysis of key genes, microRNAs and long non‐coding RNAs in hepatocellular carcinoma

**DOI:** 10.1002/2211-5463.12483

**Published:** 2018-07-31

**Authors:** Baoqi Shi, Xuejun Zhang, Lumeng Chao, Yu Zheng, Yongsheng Tan, Liang Wang, Wei Zhang

**Affiliations:** ^1^ Department of Intervention Inner Mongolia People's Hospital Hohhot China

**Keywords:** hepatocellular carcinoma, long non‐coding RNA, miRNA, mRNA, TCGA

## Abstract

Human hepatocellular carcinoma (HCC) is a common aggressive cancer whose molecular mechanism remains elusive. We aimed to identify the key genes, microRNAs (miRNAs) and long non‐coding RNAs (lncRNAs) involved with HCC. We obtained mRNA, miRNA and lncRNA profiles for HCC from The Cancer Genome Atlas and then identified differentially expressed mRNAs (DEmRNAs), miRNAs (DEmiRNAs) and lncRNAs (DElncRNAs). We performed functional annotation of DEmRNAs and then constructed HCC‐specific DEmiRNA–DEmRNA, DEmiRNA–DElncRNA and DElncRNA–DEmiRNA–DEmRNA interaction networks. We searched for nearby target *cis*‐DEmRNAs of DElncRNAs and performed receiver operating characteristic and survival analyses. A total of 1239 DEmRNAs, 33 DEmiRNAs and 167 DElncRNAs in HCC were obtained. Retinol metabolism [false discovery rate (FDR) = 7.02 × 10^−14^] and metabolism of xenobiotics by cytochrome P450 (FDR = 7.30 × 10^−11^) were two significantly enriched pathways in HCC. We obtained 545 DEmiRNA–DEmRNA pairs that consisted of 258 DEmRNAs and 28 DEmiRNAs in HCC. mir‐424, miR‐93 and miR‐3607 are three hub DEmiRNAs of the HCC‐specific DEmiRNA–DEmRNA interaction network. HAND2‐AS1/ENSG00000232855–miR‐93–*LRAT/RND3*, ENSG00000232855–miR‐877–*RCAN1* and ENSG00000232855–miR‐224–*RND3* interactions were found in the HCC‐specific DElncRNA–DEmiRNA–DEmRNA interaction network. A total of three DElncRNA–nearby target DEmRNA pairs (HCG25–*KIFC1*, LOC105378687–*CDC20* and LOC101927043–*EPCAM*) in HCC were obtained. Diagnostic and prognostic values of several selected DElncRNAs, DEmRNAs and DEmiRNAs for HCC were assessed. Our study identified several DEmRNAs, DEmiRNAs and DElncRNAs with great diagnostic or prognostic value for HCC, which may facilitate studies into the molecular mechanisms, and development of potential biomarkers and therapeutic target sites for HCC.

**Abbreviations**
AUC: area under the ROC curve
CDC20: cell division cycle protein 20
DElncRNA: differentially expressed lncRNA
DEmiRNA: differentially expressed miRNA
DEmRNA: differentially expressed mRNA
EEC: endometrioid endometrial carcinoma
EPCAM: epithelial cell adhesion molecule
FC: fold change
FDR: false discovery rate
GO: Gene Ontology
HCC: hepatocellular carcinoma
HSC: hepatic stellate cell
KEGG: Kyoto Encyclopedia of Genes and Genomes
KIFC1: kinesin family member C1
lncRNA: long non‐coding RNA
LRAT: lecithin retinol acyltransferase
miRNA: microRNA
RCAN1: regulator of calcineurin 1
RND3: Rho family GTPase 3
TCGA: The Cancer Genome Atlas

Human hepatocellular carcinoma (HCC) is the fifth most common cancer as well as the third leading cause of cancer‐related mortality worldwide 1. It is a highly aggressive cancer that is characterized by fast infiltrating growth, early metastasis, high‐grade malignancy and poor prognosis 2. Only around 10–20% of patients with HCC are diagnosed at the early stage due to lack of effective diagnostic approaches 3, 4. Moreover, the long‐term overall survival rate remains rather low despite various therapeutic strategies for HCC having been developed 5. Hence, it is crucial to elucidate the mechanism and develop accurate diagnostic biomarkers and effective therapeutic strategies for HCC.

Previous studies have identified risk factors of HCC such as chronic infection with hepatitis B virus and hepatitis C virus, hepatocirrhosis induced by alcohol, other chronic inflammatory‐related factors and hepatic regenerative changes 6, 7, 8. However, the molecular mechanism of HCC remains largely unknown. Aberrantly expressed genes such as *RND3*,* LRAT*,* ECHS1*,* ACAA1*,* MT2A* and *MYC* have been demonstrated to be associated with the pathogenesis of HCC 9, 10, 11. In addition, accumulated evidence has demonstrated that aberrantly expressed microRNAs (miRNAs), such as miR‐21, miR‐93, miR‐424, miR‐181b, miR‐221, miR‐222 and miR‐122, were associated with the development and progression of HCC 12, 13, 14. Long non‐coding RNAs (lncRNAs) are a class of conserved non‐protein‐coding RNAs with more than 200 nucleotides that are broadly distributed in the human genome 15. They involve many biological processes and could regulate gene expression in *cis* or in *trans* by diverse mechanisms 16. They were reported to play key roles in various cancers such as colorectal cancer, breast cancer and HCC 17, 18, 19. However, only a handful of HCC‐associated lncRNAs, such as HULC, HOTAIR, MEG3, MVIH and MTIDP, have been investigated 17, 18. To better understand the mechanism of HCC, it is crucial to identify key genes, miRNAs and lncRNAs in HCC. Moreover, many previous studies focused on revealing the functions of each individual gene, miRNA and lncRNA in the process of HCC, and hence mechanistic relationships among them remain largely unknown.

In this study, comprehensive analysis of mRNA, miRNA and lncRNA profiling data of HCC from The Cancer Genome Atlas (TCGA) was performed. We identified differentially expressed mRNAs (DEmRNAs), miRNAs (DEmiRNAs) and lncRNAs (DElncRNAs) in HCC. Based on bioinformatics analysis, interactions among DEmRNAs, DEmiRNAs and DElncRNAs were analyzed. Receiver operating characteristic (ROC) and survival analyses were performed to access the diagnostic and prognostic value of selected DElncRNAs, DEmRNAs and DEmiRNAs for HCC. Our study may provide new clues for exploring molecular mechanisms of HCC and developing HCC‐associated diagnostic and therapeutic approaches.

## Materials and methods

### mRNA, miRNA and lncRNA profiles of HCC in TCGA

The Cancer Genome Atlas is a central bank for multidimensional data of various cancers at DNA, RNA and protein levels. In this study, the clinical information of patients with HCC was downloaded from TCGA data portal (http://tcga-data.nci.nih.gov/). rsem‐normalized mRNA and lncRNA expression profiles (Level 3‐IlluminaHiseq_RNASeqV2 data) and miRNA expression profile (Level 3‐IlluminaHiSeq‐miRNASeq data) between HCC and adjacent normal tissues were downloaded from TCGA data portal (http://tcga-data.nci.nih.gov/) as well.

### DEmRNAs, DEmiRNAs and DElncRNAs in HCC compared with adjacent tissues

Before identifying the DEmRNAs, DEmiRNAs and DElncRNAs between HCC and normal tissues, we firstly filtered the difficultly detected miRNAs, mRNAs and lncRNAs (miRNAs, mRNAs and lncRNAs with read count value = 0 in more than 10% of HCC cases or in more than 10% of normal tissues).

Then, based on the read count of each sample, the DEmRNAs and DEmiRNAs in HCC compared with adjacent tissues were calculated with the R‐bioconductor package deseq2 20 with false discovery rate (FDR) < 0.01 and abs [log2 fold change (FC)] > 1.5. Based on the BAM files, we used reads per kilobase per million reads (RPKM) to quantify the expression levels of lncRNAs. Student's *t* test was performed to obtain *P* values. Using the Benjamini and Hochberg method, multiple comparisons were performed to obtain the FDR 21. The threshold for the DElncRNAs was FDR < 0.01 and abs (log2 FC) > 1.5 as well.

### Functional annotation of DEmRNAs between HCC and normal tissues

Functional annotation, including Gene Ontology (GO) classification and Kyoto Encyclopedia of Genes and Genomes (KEGG) pathway enrichment analysis of DEmRNAs between HCC and normal tissues, was conducted using online software genecodis (http://genecodis.cnb.csic.es/analysis). Statistical significance was defined as FDR < 0.05.

### HCC‐specific DEmiRNA–DEmRNA interaction network

Firstly, pairwise Pearson correlation coefficients between DEmRNAs and DEmiRNAs were calculated. DEmiRNA–DEmRNA pairs with *P* < 0.05 and *r* < 0 were served as significant negative DEmiRNA–DEmRNA co‐expression pairs. Then, the putative targeted DEmRNAs of DEmiRNAs were predicted by six bioinformatic algorithms (rna22, miranda, mirdb, mirwalk, pictar2 and targetscan) of mirwalk2.0 (http://zmf.umm.uni-heidelberg.de/apps/zmf/mirwalk2/mir-mir-self.html). Targets recorded by ≥ 4 algorithms were served as target DEmRNAs of DEmiRNAs. The confirmed target DEmRNAs of DEmiRNAs were obtained by mirwalk2.0 http://zmf.umm.uni-heidelberg.de/apps/zmf/mirwalk2/mir-mir-self.html as well. Finally, DEmiRNA–DEmRNA co‐expression pairs were obtained whose DEmRNA was not only negatively co‐expressed with DEmiRNAs but also the predicted targets of this DEmiRNA with ≥ 4 algorithms or confirmed targets of this DEmiRNA. Based on these DEmiRNA–DEmRNA pairs, the DEmiRNA–DEmRNA interaction network was constructed and visualized using cytoscape software (http://www.cytoscape.org/).

### HCC‐specific DElncRNA–DEmiRNA interaction network

Firstly, pairwise Pearson correlation coefficients between DElncRNAs and DEmiRNAs were calculated. DElncRNA–DEmiRNA pairs with *P* < 0.05 and *r* < 0 were served as significant negative DElncRNA–DEmiRNA co‐expression pairs. Then, the putative targeted DElncRNAs of DEmiRNAs were predicted by miRWalk of mirwalk2.0 http://zmf.umm.uni-heidelberg.de/apps/zmf/mirwalk2/mir-mir-self.html. Finally, DElncRNA–DEmiRNA pairs whose DElncRNA was not only negatively co‐expressed with DEmiRNAs but also the predicted targets of this DEmiRNA by miRWalk were obtained. Based on these DElncRNA–DEmiRNA pairs, the DElncRNA–DEmiRNA interaction network was constructed and visualized using cytoscape softwarehttp://www.cytoscape.org/.

### HCC‐specific DElncRNA–DEmiRNA–DEmRNA interaction network

The HCC‐specific DElncRNA–DEmiRNA–DEmRNA interaction network was constructed by merging the HCC‐specific DEmiRNA–DEmRNA interaction network and DElncRNA–DEmiRNA interaction network based on the common DEmiRNAs.

### Nearby targeted DEmRNAs of DElncRNAs in HCC

To identify the target DEmRNAs of DElncRNAs by *cis*‐regulatory effects, we searched the DEmRNAs transcribed within a 200‐kb window up‐ or downstream of DElncRNAs that were served as nearby *cis*‐targeted DEmRNAs of DElncRNAs.

### ROC analysis

In order to access the diagnostic value of DElncRNAs, DEmRNAs and DEmiRNAs for HCC, respectively, the proc package was used to calculate ROC, and the area under the ROC curve (AUC) was further calculated. When AUC value was greater than 0.8, the DElncRNAs/DEmRNAs/DEmiRNAs were considered capable of distinguishing patients with HCC and normal controls with excellent specificity and sensitivity.

### Survival analysis

Using survival (https://cran.r-project.org/web/packages/survival/index.html) in R, the prognostic value of selected DElncRNAs, DEmRNAs and DEmiRNAs for patients with HCC was analyzed.

## Results

### DEmRNAs, DEmiRNAs and DElncRNAs in HCC

Data for a total of 377 patients with HCC were downloaded from TCGA data portal. From these there were obtained the mRNA expression profile of HCC tissues of 371 patients with HCC and 50 adjacent tissues, the miRNA expression profile of HCC tissues of 372 patients with HCC and 50 adjacent tissues and the lncRNA expression profile of HCC tissues of 200 patients with HCC and 50 adjacent tissues.

**Figure 1:**
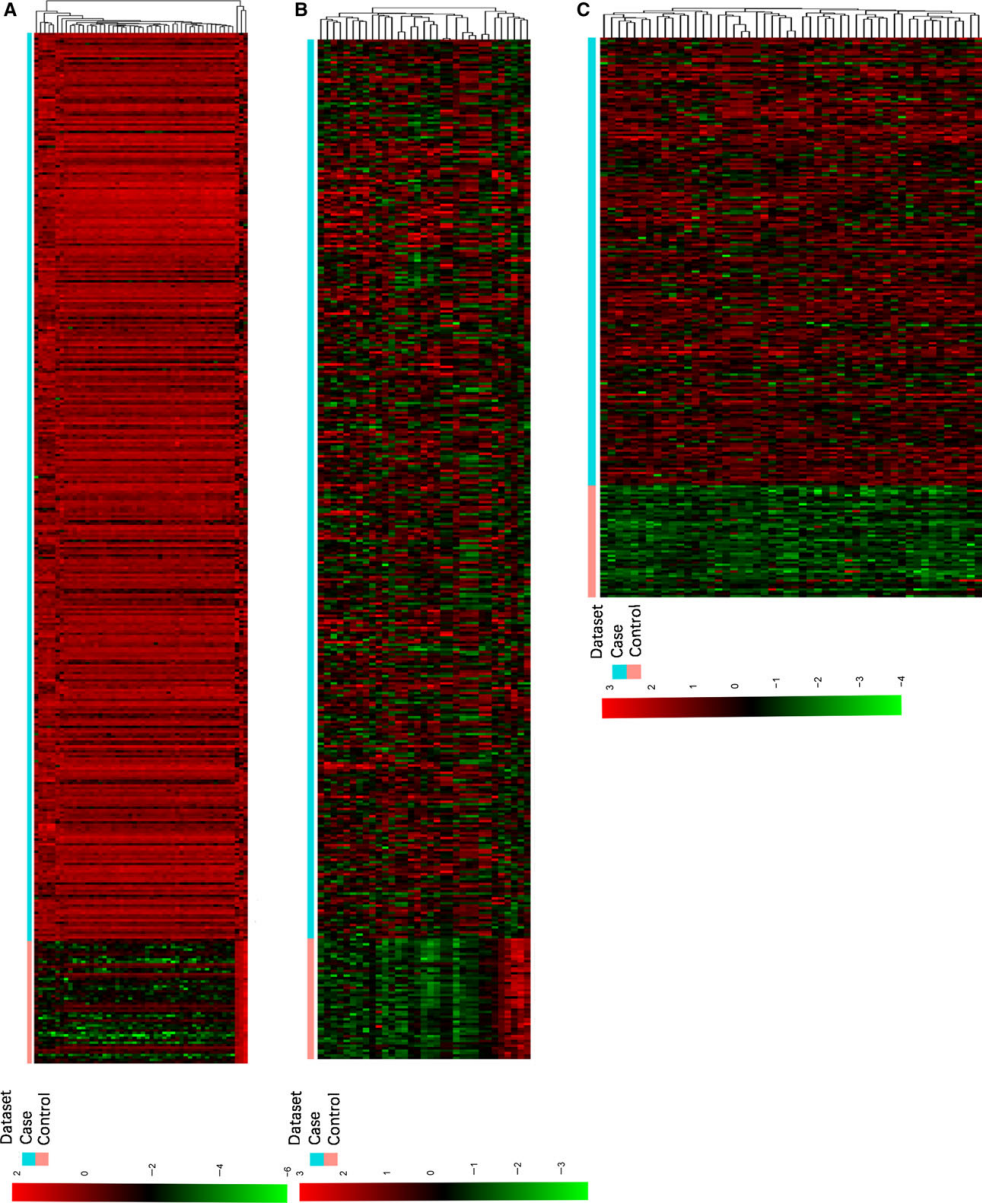
Hierarchical clustering analysis of the DEmRNAs, DEmiRNAs and DElncRNAs in HCC and adjacent normal tissues. Rows and columns represent samples and DEmRNAs, DEmiRNAs, and DElncRNAs, respectively. Red and green represent up‐ and downregulation, respectively. Case: HCC tissues; control: adjacent normal tissues. (A) Hierarchical clustering analysis of the top 50 DEmRNAs in HCC; (B) hierarchical clustering analysis of the DEmiRNAs in HCC; (C) hierarchical clustering analysis of the top 50 DElncRNAs in HCC.

After filtering the difficultly detected miRNAs, mRNAs and lncRNAs, a total of 311 miRNAs, 14 607 mRNAs and 2152 lncRNAs were retained for analysis. A total of 1239 DEmRNAs (865 of them upregulated and 374 of them downregulated), 33 DEmiRNAs (29 upregulated and four downregulated) and 167 DElncRNAs (165 upregulated and two downregulated) in HCC were obtained. A heat‐map of DEmRNAs, DEmiRNAs and DElncRNAs in HCC is displayed in Fig. 1. The top 10 up‐ and downregulated DEmRNAs and DEmiRNAs, and the top 20 DElncRNAs between HCC and normal tissues are displayed in Tables 1, 2, 3, respectively.

**Table 1:** Top 10 up‐ and downregulated DEmRNAs between HCC and normal tissues

Gene ID	Symbol	Log FC	*P* value	FDR	Regulation
1033	*CDKN3*	4.40	1.02E‐106	1.50E‐102	Up
83540	*NUF2*	4.77	9.25E‐102	6.76E‐98	Up
1306	*COL15A1*	4.28	2.30E‐100	1.12E‐96	Up
83483	*PLVAP*	2.89	1.82E‐98	6.66E‐95	Up
24137	*KIF4A*	4.35	7.46E‐96	2.18E‐92	Up
29089	*UBE2T*	3.43	1.02E‐94	2.47E‐91	Up
1063	*CENPF*	3.94	3.91E‐93	8.17E‐90	Up
3833	*KIFC1*	3.96	5.94E‐93	1.08E‐89	Up
9833	*MELK*	4.17	2.48E‐90	4.02E‐87	Up
11004	*KIF2C*	4.38	5.64E‐90	8.24E‐87	Up
11093	*ADAMTS13*	−2.90	1.25E‐77	4.95E‐75	Down
170392	*OIT3*	−3.56	2.08E‐73	6.19E‐71	Down
64651	*CSRNP1*	−2.24	6.54E‐60	1.02E‐57	Down
1893	*ECM1*	−3.02	2.44E‐54	3.05E‐52	Down
1827	*RCAN1*	−2.39	9.08E‐53	1.05E‐50	Down
5199	*CFP*	−3.33	6.69E‐52	7.40E‐50	Down
390	*RND3*	−2.47	2.22E‐51	2.35E‐49	Down
83854	*ANGPTL6*	−3.02	7.92E‐49	7.51E‐47	Down
7538	*ZFP36*	−2.15	6.23E‐47	5.42E‐45	Down
9227	*LRAT*	−3.22	2.35E‐45	1.82E‐43	Down

**Table 2:** DEmiRNAs between HCC and normal tissues

DEmiRNA	Log FC	*P* value	FDR	Regulation
hsa‐mir‐424	−2.21	5.92E‐61	1.84E‐58	Down
hsa‐mir‐10b	3.59	6.75E‐59	1.05E‐56	Up
hsa‐mir‐21	1.84	6.65E‐55	6.89E‐53	Up
hsa‐mir‐93	1.71	2.22E‐51	1.73E‐49	Up
hsa‐mir‐589	1.58	1.89E‐50	1.18E‐48	Up
hsa‐mir‐224	3.26	6.52E‐47	3.38E‐45	Up
hsa‐mir‐183	3.86	2.29E‐46	1.02E‐44	Up
hsa‐mir‐1269	5.56	8.76E‐46	3.41E‐44	Up
hsa‐mir‐96	3.75	3.42E‐40	1.18E‐38	Up
hsa‐mir‐500a	1.60	4.26E‐40	1.32E‐38	Up
hsa‐mir‐182	3.37	5.13E‐39	1.45E‐37	Up
hsa‐mir‐452	2.50	2.09E‐36	5.40E‐35	Up
hsa‐mir‐221	1.57	5.83E‐30	1.13E‐28	Up
hsa‐mir‐217	4.00	1.11E‐27	1.93E‐26	Up
hsa‐mir‐1180	1.77	8.59E‐27	1.34E‐25	Up
hsa‐mir‐9‐1	3.23	1.31E‐26	1.85E‐25	Up
hsa‐mir‐9‐2	3.22	1.49E‐26	2.02E‐25	Up
hsa‐mir‐196b	3.28	5.01E‐26	6.00E‐25	Up
hsa‐mir‐1266	2.06	6.79E‐26	7.54E‐25	Up
hsa‐mir‐3200	2.54	3.87E‐23	3.89E‐22	Up
hsa‐mir‐877	1.80	1.04E‐21	9.26E‐21	Up
hsa‐mir‐3677	1.65	5.26E‐21	4.42E‐20	Up
hsa‐mir‐18a	1.67	7.16E‐20	5.57E‐19	Up
hsa‐mir‐216a	3.45	1.48E‐19	1.10E‐18	Up
hsa‐mir‐19a	1.55	2.90E‐19	2.05E‐18	Up
hsa‐mir‐3607	−1.65	1.06E‐16	6.12E‐16	Down
hsa‐mir‐1274b	−1.52	1.83E‐15	9.81E‐15	Down
hsa‐mir‐508	2.19	6.42E‐15	3.33E‐14	Up
hsa‐mir‐937	1.68	1.39E‐13	6.17E‐13	Up
hsa‐mir‐1226	1.72	1.87E‐13	7.98E‐13	Up
hsa‐mir‐3648	−1.52	2.81E‐11	1.03E‐10	Down
hsa‐mir‐431	1.53	3.05E‐08	8.54E‐08	Up
hsa‐mir‐483	1.79	2.02E‐05	4.29E‐05	Up

**Table 3:** Top 20 DElncRNAs between HCC and normal tissues

ENSG	ID	Symbol	Log FC	*P* value	FDR	Regulation
ENSG00000267080	339201	ASB16‐AS1	1.52	2.51E‐38	1.80E‐35	Up
ENSG00000212694	338799	LINC01089	2.21	5.18E‐36	1.59E‐33	Up
ENSG00000206573	440944	THUMPD3‐AS1	1.67	1.00E‐32	1.32E‐30	Up
ENSG00000232995	8490	RGS5	1.99	1.05E‐32	1.32E‐30	Up
ENSG00000249592	100129917	LOC100129917	1.66	1.32E‐32	1.58E‐30	Up
ENSG00000234608	51275	MAPKAPK5‐AS1	1.54	1.44E‐32	1.63E‐30	Up
ENSG00000228288	100506696	PCAT6	2.34	5.79E‐31	4.29E‐29	Up
ENSG00000228265	101926888	RALY‐AS1	1.52	3.47E‐30	2.07E‐28	Up
ENSG00000213742	102724826	ZNF337‐AS1	1.68	3.29E‐30	2.07E‐28	Up
ENSG00000224424	100506637	PRKAR2A‐AS1	2.26	5.79E‐30	3.20E‐28	Up
ENSG00000172965	541471	MIR4435‐2HG	2.53	6.70E‐30	3.52E‐28	Up
ENSG00000234912	654434	SNHG20	1.67	2.32E‐28	9.43E‐27	Up
ENSG00000233527	101927599	ZNF529‐AS1	1.69	5.34E‐28	1.89E‐26	Up
ENSG00000228106	102724017	LOC102724017	1.56	1.50E‐27	4.82E‐26	Up
ENSG00000250988	100505616	SNHG21	1.78	2.56E‐27	7.66E‐26	Up
ENSG00000226696	104355426	LENG8‐AS1	2.19	3.76E‐27	1.11E‐25	Up
ENSG00000186615	100129075	KTN1‐AS1	1.79	1.13E‐26	2.98E‐25	Up
ENSG00000198468	642946	FLVCR1‐AS1	2.24	1.18E‐26	3.06E‐25	Up
ENSG00000232940	414765	HCG25	2.54	1.40E‐26	3.58E‐25	Up
ENSG00000234432	100129484	LOC100129484	1.88	1.95E‐26	4.60E‐25	Up

### Functional annotation of DEmRNAs between HCC and normal tissues

**Figure 2:**
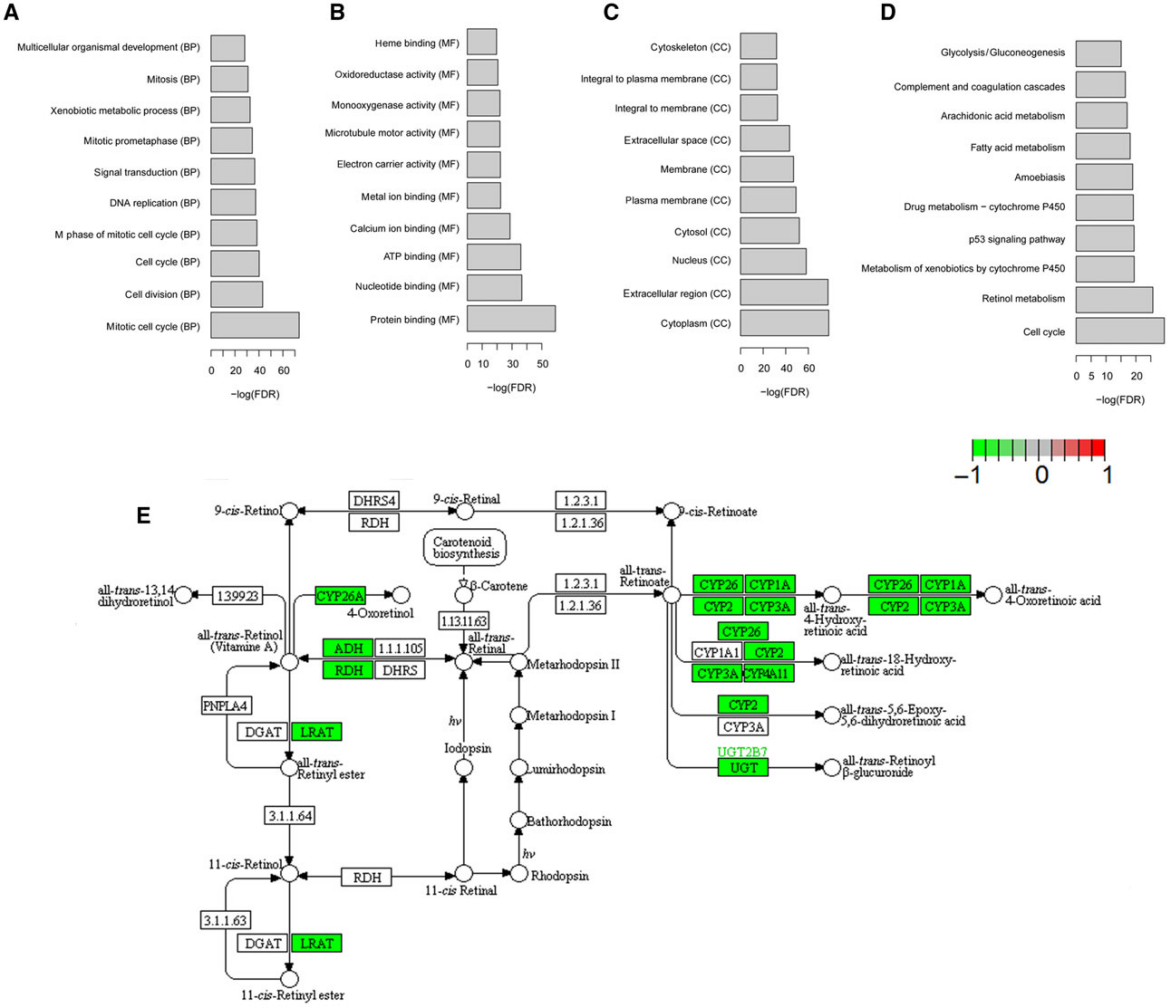
Functional annotation of DEmRNAs between HCC and normal tissues. (A–D) The significantly enriched biological process (A), molecular function (B), cellular component (C) and KEGG pathways (D) for DEmRNAs between HCC and normal tissues. The *x*‐axis shows −log FDR and the *y*‐axis shows GO terms or KEGG pathways. (E) The pathway of retinol metabolism. The red and green rectangles represent the particles that are regulated by up‐ and downregulated DEmRNAs, respectively, between HCC and normal tissues.

Functional annotation of DEmRNAs between HCC and normal tissues indicated that mitotic cell cycle (FDR = 4.56 × 10^−36^), protein binding (FDR =  2.16 × 10^−26^), and cytoplasm (FDR = 1.25 × 10^−34^) were significantly enriched GO terms (Fig. 2A–C). Retinol metabolism (FDR = 7.02 × 10^−14^) and metabolism of xenobiotics by cytochrome P450 (FDR = 7.30 × 10^−11^) were two significantly enriched pathways (Fig. 2D,E).

### HCC‐specific DEmiRNA–DEmRNA interaction network

**Figure 3:**
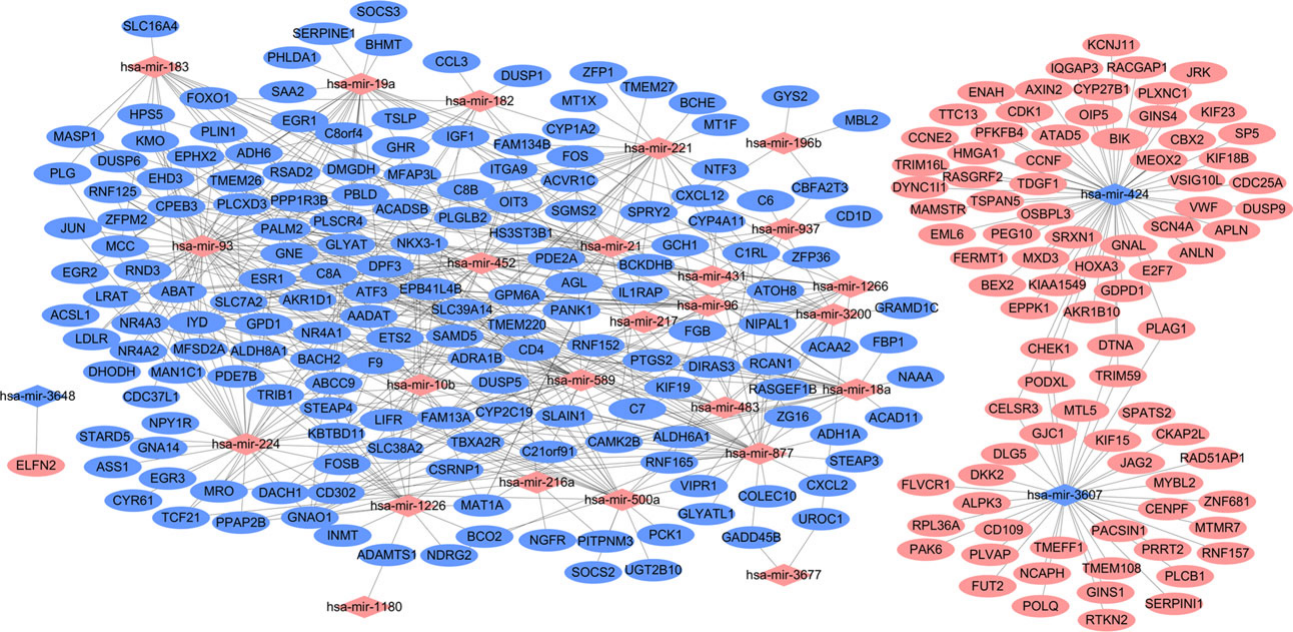
Hepatocellular carcinoma‐specific DEmiRNA–DEmRNA interaction network. Rhombuses and ellipses represent DEmiRNAs and DEmRNAs, respectively. Red and blue represent up‐ and downregulation, respectively.

Firstly, we obtained 7996 negative DEmiRNA–DEmRNA co‐expression pairs with *P* < 0.05 and *r* < 0. Then, a total of 1142 DEmiRNA‐target DEmRNA pairs with predicted ≥ 4 algorithms were obtained. Finally, 545 DEmiRNA–DEmRNA pairs were obtained whose DEmRNA was not only negatively co‐expressed with DEmiRNAs but also the predicted targets of this DEmiRNA with ≥ 4 algorithms. These 545 DEmiRNA–DEmRNA pairs consisted of 258 DEmRNAs (88 upregulated and 170 downregulated) and 28 DEmiRNAs (25 upregulated and three downregulated) in HCC. The HCC‐specific DEmiRNA–DEmRNA interaction network is displayed in Fig. 3. mir‐424 (degree = 56), miR‐93 (degree = 51), and miR‐3607 (degree = 37) are three hub DEmiRNAs.

### HCC‐specific DElncRNA–DEmiRNA interaction network

**Figure 4:**
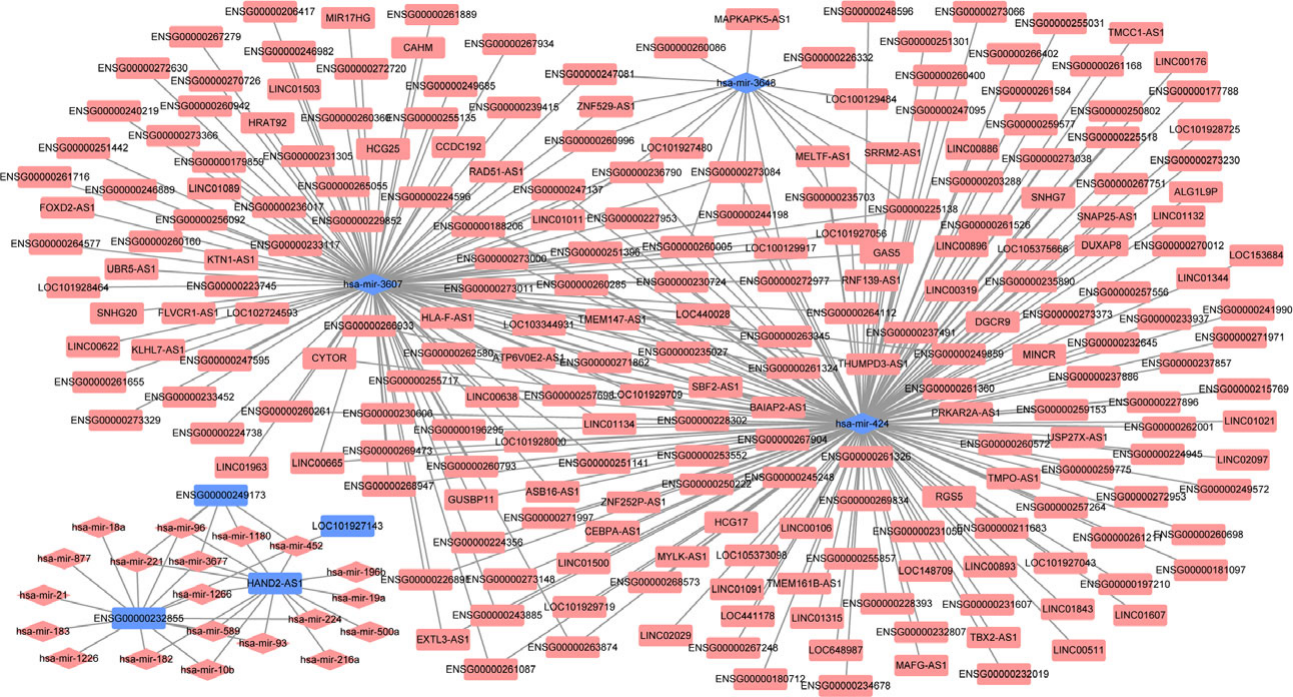
Hepatocellular carcinoma‐specific DEmiRNA–DElncRNA interaction network. Rhombuses and rectangles represent DEmiRNAs and DElncRNAs, respectively. Red and blue represent up‐ and downregulation, respectively.

Firstly, we obtained 1258 negative DElncRNA–DEmiRNA co‐expression pairs with *P* < 0.05 and *r* < 0. Then, a total of 7090 DEmiRNA‐target DElncRNA pairs were obtained by mirwalk. Finally, we obtained 342 DEmiRNA–DElncRNA pairs whose DElncRNA was not only negatively coexpressed with DEmiRNA but also the predicted targets of this DEmiRNA based on mirwalk. The HCC‐specific DElncRNA–DEmiRNA interaction network consisted of 260 nodes and 342 edges (Fig. 4). miR‐424 (degree = 171) and miR‐3648 (degree = 11) were hub DEmiRNAs of an HCC‐specific DElncRNA–DEmiRNA interaction network.

### HCC‐specific DElncRNA–DEmiRNA–DEmRNA interaction network

**Figure 5:**
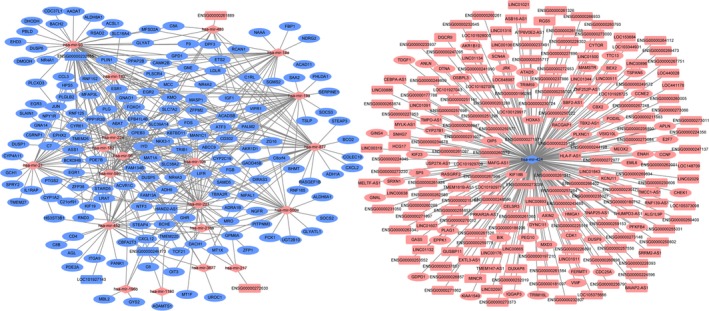
Hepatocellular carcinoma‐specific DEmiRNA–DElncRNA–DEmRNA interaction network. Rectangles, rhombuses and ellipses represent DElncRNAs, DEmiRNAs and DEmRNAs, respectively. Red and blue represent up‐ and downregulation, respectively.

The HCC‐specific DElncRNA–DEmiRNA–DEmRNA interaction network consisted of 417 nodes and 651 edges. HAND2‐AS1/ENSG00000232855–miR‐93–lecit hin retinol acyltransferase (LRAT)/Rho family GTPase 3 (RND3), ENSG00000232855–miR‐877–regulator of calcineurin 1 (RCAN1) and ENSG00000232855–miR‐224–*RND3* interactions were found in this HCC‐specific DElncRNA–DEmiRNA–DEmRNA interaction network (Fig. 5).

### Nearby targeted DEmRNAs of DElncRNAs in HCC

A total of three DEmRNAs transcribed within a 200‐kb window up‐ or downstream of three DElncRNAs in HCC were obtained. HCG25–*kinesin family member C1* (*KIFC1*), LOC105378687–*cell division cycle protein 20* (*CDC20*) and LOC101927043–*epithelial cell adhesion molecule* (*EPCAM*) are three DElncRNA–nearby target DEmRNA pairs (Table 4).

**Table 4:** DElncRNA‐nearby targeted DEmRNA pairs in HCC. Chr, chromosome

lncRNA					Nearby targeted mRNA		
Chr	lncRNA ENSG	lncRNA symbol	Start − 200 kb	End + 200 kb	mRNA symbol	Start	End
chr6	ENSG00000232940	HCG25	33049534	33454989	*KIFC1*	33391536	33409924
chr1	ENSG00000234694	LOC105378687	43154684	43558658	*CDC20*	43358955	43363203
chr2	ENSG00000234690	LOC101927043	46992405	47545074	*EPCAM*	47345158	47387601

### ROC analysis

**Figure 6:**
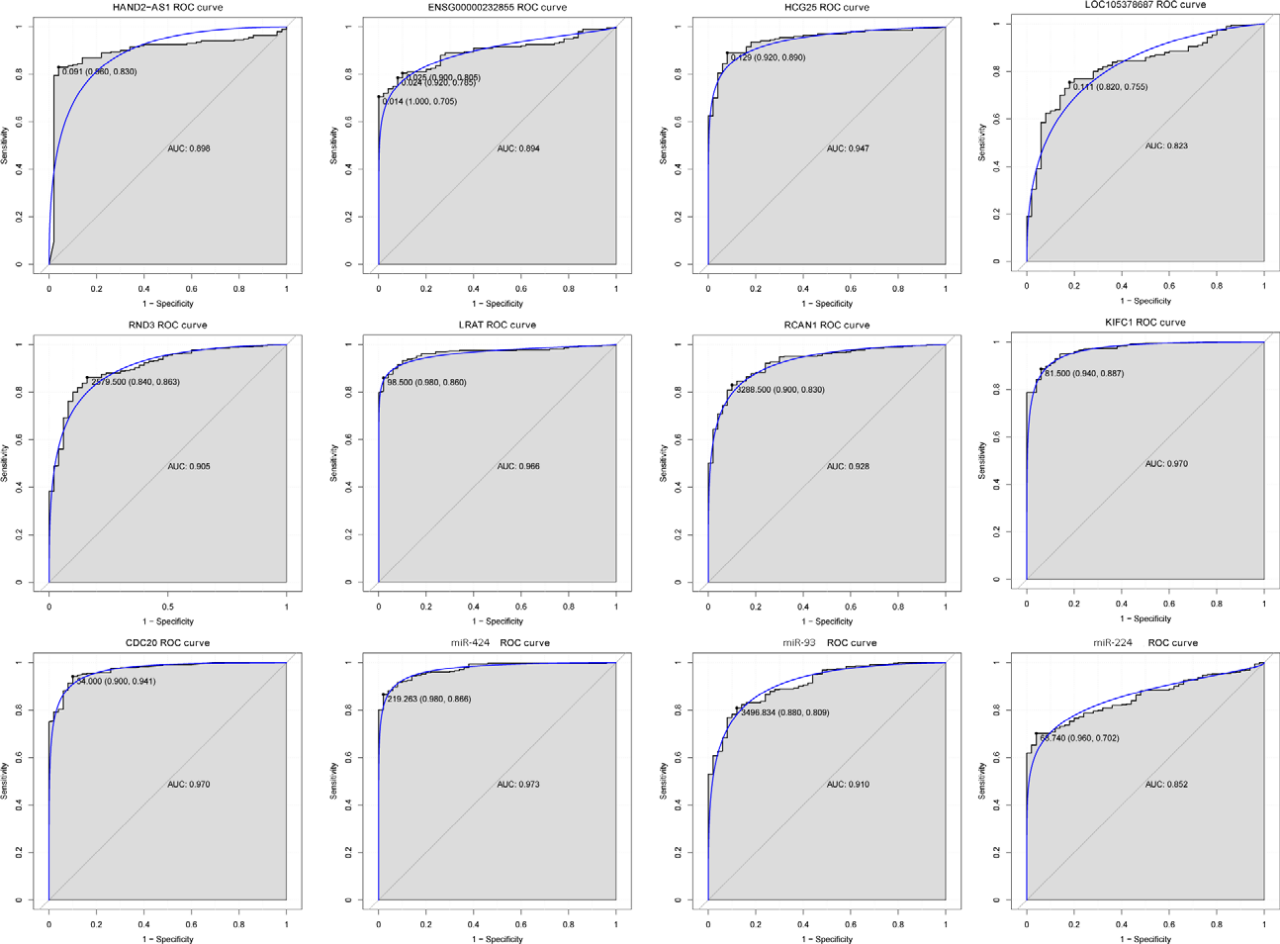
ROC analysis of selected DEmRNAs, DEmiRNAs and DElncRNAs. ROC curves were used to show the diagnostic value of selected DElncRNAs, DEmRNAs and DEmiRNAs for HCC with sensitivity and specificity. The *x*‐axis indicates 1 − specificity, and *y*‐axis indicates sensitivity. Names of the DElncRNAs, DEmRNAs and DEmiRNAs are displayed above the ROC curve.

ROC curve analysis was performed to evaluate the diagnostic value of five DElncRNAs (HAND2‐AS1, ENSG00000232855, HCG25, LOC105378687 and LOC101927043), five DEmRNAs (*RND3, LART, RCAN1, KIFC1* and *CDC20*) and four DEmiRNAs (miR‐424, miR‐93, miR‐224 and miR‐877) for HCC. Except for LOC101927043 and miR‐877, the other four DElncRNAs (HAND2‐AS1, ENSG00000232855, HCG25 and LOC105378687), five DEmRNAs (*RND3, LART, RCAN1, KIFC1* and *CDC20*) and three DEmiRNAs (miR‐424, miR‐93 and miR‐224) have great diagnostic value for HCC with AUC more than 0.8 (Fig. 6).

### Survival analysis

**Figure 7:**
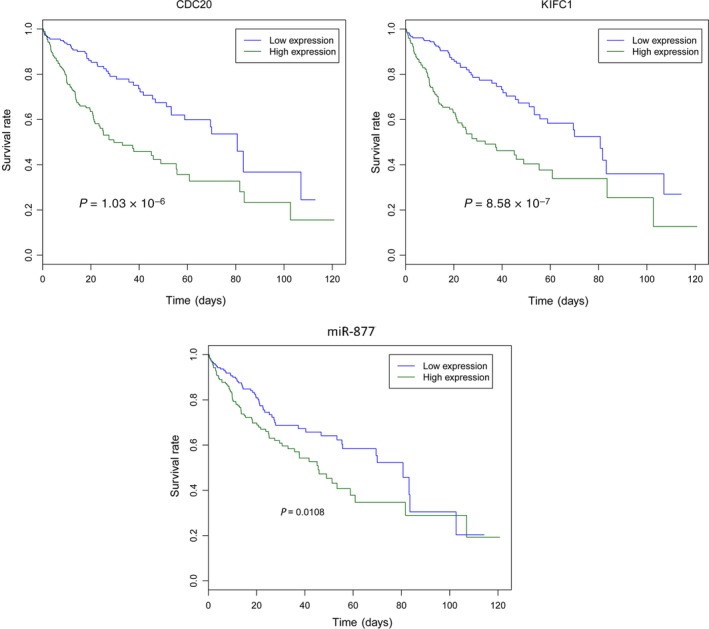
Survival analysis of selected DEmRNAs, DEmiRNAs, and DElncRNAs. Survival curves were used to show the prognostic value of selected DEmRNAs and DEmiRNAs for HCC. The *x*‐axis indicated times (days), and *y*‐axis indicated survival rate. Above the survival curves, names of DEmRNAs and DEmiRNAs were displayed. High expression of CDC20 (*P *=* *1.03 × 10^−6^), KIFC1 (*P *=* *8.58 × 10^−7^), and miR‐877 (*P *=* *0.0108) were significantly associated with lower survival rate in patients with HCC.

Survival analysis was performed to evaluate the prognostic value of five DElncRNAs (HAND2‐AS1, ENSG00000232855, HCG25, LOC105378687 and LOC101927043), five DEmRNAs (*RND3, LART, RCAN1, KIFC1* and *CDC20*) and four DEmiRNAs (miR‐424, miR‐93, miR‐224 and miR‐877) for HCC. Only two DEmRNAs (*CDC20* and *KIFC1*) and miR‐877 have prognostic value for HCC. High expression of *CDC20* (*P *=* *1.03 × 10^−6^), *KIFC1* (*P *=* *8.58 × 10^−7^) and miR‐877 (*P *=* *0.0108) was significantly associated with a lower survival rate in patients with HCC (Fig. 7).

## Discussion

In this study, we identified DEmRNAs, DEmiRNAs and DElncRNAs between HCC and normal controls from TCGA. Their interactions and potential diagnostic and prognostic value for HCC were further examined by bioinformatics analysis.

Functional annotation of DEmRNAs indicated that retinol metabolism was a significantly enriched pathway in HCC. Retinoic acids have been demonstrated to play an inhibitory role in carcinogenesis of various cancers, including HCC 22. Inhibition of retinoic acid signaling in hepatocytes provoked the development of liver cancer in transgenic mice 23. Metabolism of xenobiotics by cytochrome P450 was another significantly enriched pathway in HCC. This is a typical liver‑function‑specific pathway and has been indicated to play crucial roles in HCC 24. The members of the cytochrome P450 (CYP) family have frequently been found to be involved in various biological processes that were found to be dysregulated in liver cancer 25. Hence, DEmRNAs enriched in these two pathways might be regulators in HCC, and this needs further research.

Our study provided evidence for several HCC‐related mRNAs identified in previous studies. Moreover, their functions in HCC were further studied by the interaction of DElncRNAs and DEmiRNAs with them. Based on the present study, miR‐424, miR‐93 and miR‐224 are three hub miRNAs of an HCC‐specific DEmiRNA–DEmRNA interaction network and all of them have great diagnostic value for HCC, suggesting their importance in HCC. Upregulated miR‐93 has been found in patients with HCC in previous studies, which is consistent with the present study 14. Increased miR‐93 was associated with cell migration and invasion of HCC and serves as a potential marker of poor 5‐year overall survival of patients with HCC 14, 26. Based on our DEmiRNA–DEmRNA interaction network, miR‐424, miR‐93 and miR‐224 had 56, 51 and 34 targeted DEmRNAs in HCC, respectively. RND3 was a shared target of both miR‐93 and miR‐224. LRAT was another target of miR‐93. Both *RND3* and *LRAT* are two downregulated DEmRNAs derived from the top 10 downregulated DEmRNAs and have great diagnostic value for HCC.

Previous studies have indicated that both *RND3* and *LRAT* are HCC‐related genes. RND3 is a member of the RND subfamily of the Rho GTPase family. *RND3* was significantly downregulated in HCC cell lines and tissues. HCC cell growth could be inhibited by knockdown of RND3 10. *RND3* was speculated to regulate a switch to attenuate cell growth and favor cell invasion and serve as a potential metastasis suppressor gene in HCC 10. Retinoid is mainly stored in the liver in the form of retinyl ester in lipid droplets. Hepatic stellate cells (HSCs) serve as the major cells of retinoid storage within the liver 27. Lack of retinoid‐containing lipid droplets of HSCs has been observed in the development of liver disease leading to HCC 27. As the sole enzyme that conducts the synthesis of hepatic retinyl ester, LRAT may play a key role in the pathogenesis of HCC 11. Our study found that *LRAT* was downregulated in patients with HCC, which was consistent with a previous study 28. Taken together, miR‐93–RND3/LRAT and miR‐224–RND3 interactions may play crucial roles in HCC.

lncRNAs were reported to bind to miRNA and act as sponges for miRNAs 29. By sharing common miRNA binding sites with mRNA targets, lncRNAs sequester and compete with miRNA to inhibit miRNA function and alleviate mRNA repression 30. In the present study, we constructed the lncRNA–miRNA–mRNA interaction network based on the shared common miRNAs.

Two downregulated lncRNAs (HAND2‐AS1 and ENSG00000232855) with great diagnostic value for HCC were shared targets of both miR‐93 and miR‐244. HAND2‐AS1 transcribed antisense adjacent to heart and neural crest derivatives expressed 2 (HAND2) in chromosome 4q33‐34 31. HAND2‐AS1 was reported to play an inhibiting role in migration and invasion of endometrioid endometrial carcinoma (EEC) cells by inactivating neuromedin U 31. Downregulated HAND2‐AS1 has been found in EEC tissues 31. Moreover, HAND2‐AS1 was closely associated with tumor grade, lymph node metastasis and recurrence of EEC patients and serves as a potential prognostic biomarker 31. A recent study indicated that HAND2‐AS1 was also downregulated in HCC tissues, which was associated with migration of HCC cells 32. In the present study, HAND2‐AS1 was downregulated in HCC, which provided evidence in support of the previous study. We speculate that HAND2‐AS1 might be involved with the process of HCC by inhibiting miR‐93 and miR‐244 and competing with their targets such as LRAT and RND3. Like miR‐93, ENSG00000232855 was speculated to play roles in HCC as well.

Additionally, ENSG00000232855 was a target of another HCC‐related miRNA, miR‐877. A previous study indicated that miR‐877 plays a regulating role in cell proliferation, apoptosis and the cell cycle of HCC 33. In this study, we highlighted the prognostic value of miR‐877 for HCC. Considering targeted DEmRNAs of miR‐877, *RCAN1* was a downregulated DEmRNA derived from the top 10 downregulated DEmRNAs in HCC in the present study. Downregulation of *RCAN1* has been found in HCC tissues. Based on the experiments *in vitro*, RCAN1 has an inhibitory role in cell proliferation, migration and invasion of HCC cells 34. ENSG00000232855–miR‐877–*RCAN1* interaction was speculated to play key roles in the process of HCC.

In addition, we obtained three DElncRNA–nearby target DEmRNA pairs, namely HCG25–*KIFC1*, LOC105378687–*CDC20* and LOC101927043–*EPCAM*. *KIFC1* was widely overexpressed in various cancers such as breast cancer, non‐small‐cell lung cancer and gastric cancer, and was reported to be involved with the development and prognosis of cancers 35, 36, 37. A recent study found that overexpressed *KIFC1* was found in HCC and was associated with shorter overall survival time of patients with HCC 38. Upregulated *KIFC1* was also found in HCC with both diagnostic and prognostic value for HCC in our study, which provided evidence in support of the previous study. There is no study report on the association between HCC and HCG25. *KIFC1* was a nearby target gene of HCG25 and HCG25 was significantly upregulated in HCC and has great diagnostic value for HCC. We speculate that HCG25 may regulate the process of HCC by its *cis*‐regulatory role on the expression of *KIFC1*. As one of the key genes associated with the hepatocyte cell cycle, *CDC20* has been reported to be involved with the development of HCC 39. Silencing *CDC20* could delay hepatocellular mitotic progression and inhibit HCC cell proliferation 40, 41. In this study, both diagnostic and prognostic values of *CDC20* for HCC were observed. EPCAM is a cell surface glycoprotein that serves as a marker of cancer stem cells. Upregulated *EPCAM* has been found in HCC tissues compared with normal liver tissues. Moreover, *EPCAM* was associated with shorter survival of patients with HCC. We speculate that LOC105378687 and LOC101927043 may play roles in the development of HCC by interacting with CDC20 and EPCAM, respectively.

## Conclusions

In conclusion, our study was a comprehensive analysis of key DEmRNAs, DEmiRNAs and DElncRNAs in HCC. Based on the bioinformatics analysis, several DEmRNAs, DEmiRNAs and DElncRNAs and their interactions may play important roles in the process of HCC, which has provided clues for exploring the molecular mechanisms of HCC. Moreover, diagnostic and prognostic values of several key DEmRNAs, DEmiRNAs and DElncRNAs for HCC were found in this study, which has made a contribution toward developing potential biomarkers and therapeutic target sites for HCC.

## Author contributions

BS and XZ conceived and designed the project; XZ provided support for administration; BS, YZ and LC contributed reagents, materials and analysis tools; BS, YZ and LW collected the data; BS, YT and WZ analyzed and interpreted the data; all authors wrote and approved the final manuscript.
